# Usability Evaluation of a Noninvasive Neutropenia Screening Device (PointCheck) for Patients Undergoing Cancer Chemotherapy: Mixed Methods Observational Study

**DOI:** 10.2196/37368

**Published:** 2022-08-09

**Authors:** Ganimete Lamaj, Alberto Pablo-Trinidad, Ian Butterworth, Nolan Bell, Ryan Benasutti, Aurelien Bourquard, Alvaro Sanchez-Ferro, Carlos Castro-Gonzalez, Ana Jiménez-Ubieto, Tycho Baumann, Antonia Rodriguez-Izquierdo, Elizabeth Pottier, Anthony Shelton, Joaquin Martinez-Lopez, John Mark Sloan

**Affiliations:** 1 Leuko Labs, Inc Boston, MA United States; 2 Hematology Department Hospital Universitario 12 de Octubre Madrid Spain; 3 Section of Hematology & Medical Oncology Department of Medicine Boston University School of Medicine and Boston Medical Center Boston, MA United States

**Keywords:** digital health, usability, patient-centered care, remote monitoring, decision support systems, white blood cells, diagnosis, medical device, cancer, chemotherapy, infection, white blood cell, technology

## Abstract

**Background:**

Patients with cancer undergoing cytotoxic chemotherapy face an elevated risk of developing serious infection as a consequence of their treatment, which lowers their white blood cell count and, more specifically, their absolute neutrophil count. This condition is known as neutropenia. Neutropenia accompanied by a fever is referred to as febrile neutropenia, a common side effect of chemotherapy with a high mortality rate. The timely detection of severe neutropenia (<500 absolute neutrophil count/μL) is critical in detecting and managing febrile neutropenia. Current methods rely on blood draws, which limit them to clinical settings and do not allow frequent or portable monitoring. In this study, we demonstrated the usability of PointCheck, a noninvasive device for neutropenia screening, in a simulated home environment without clinical supervision. PointCheck automatically performs microscopy through the skin of the finger to image the blood flowing through superficial microcapillaries and enables the remote monitoring of neutropenia status, without requiring venipuncture.

**Objective:**

This study aimed to evaluate the usability of PointCheck, a noninvasive optical technology for screening severe neutropenia, with the goal of identifying potential user interface, functionality, and design issues from the perspective of untrained users.

**Methods:**

We conducted a multicenter study using quantitative and qualitative approaches to evaluate the usability of PointCheck across 154 untrained participants. We used a mixed method approach to gather usability data through user testing observations, a short-answer qualitative questionnaire, and a standardized quantitative System Usability Scale (SUS) survey to assess perceived usability and satisfaction.

**Results:**

Of the 154 participants, we found that 108 (70.1%) scored above 80.8 on the SUS across all sites, with a mean SUS score of 86.1 across all sites. Furthermore, the SUS results indicated that, out of the 151 users who completed the SUS survey, 145 (96%) found that they learned how to use PointCheck very quickly, and 141 (93.4%) felt very confident when using the device.

**Conclusions:**

We have shown that PointCheck, a novel technology for noninvasive, home-based neutropenia detection, can be safely and effectively operated by first-time users. In a simulated home environment, these users found it easy to use, with a mean SUS score of 86.1, indicating an excellent perception of usability and placing this device within the top tenth percentile of systems evaluated for usability by the SUS.

**Trial Registration:**

ClinicalTrials.gov NCT04448314; https://clinicaltrials.gov/ct2/show/NCT04448314 (Hospital Universitario 12 de Octubre registration) and NCT04448301; https://clinicaltrials.gov/ct2/show/NCT04448301 (Boston Medical Center registration)

## Introduction

### Background

One of the most serious side effects of cytotoxic chemotherapy and immunotherapy is neutropenia—a decrease in neutrophils, the most common type of white blood cell (WBC) and the most important cell needed to prevent bacterial infection. The primary clinical consequence of neutropenia is an elevated risk of life-threatening bacterial infection that typically requires immediate admission to the emergency department, hospitalization, and treatment [1-3]. Every year, approximately 850,000 patients with cancer start chemotherapy treatments in the United States [4], and 140,000 (17%) [5] will endure at least one episode of febrile neutropenia (FN), or neutropenia accompanied by a fever. FN typically requires an admission of over 1 week, costing approximately US $30,000 per episode [6,7], with associated mortality rates between 7% to 10% [8]. The timely detection and awareness of severe neutropenia (ie, <500 absolute neutrophil count/µL) [9] can be crucial to prevent and manage FN in the outpatient setting [10,11] and the emergency department [12,13].

In the current standard of care, the risk of FN is evaluated by using a priori scores, such as the Multinational Association for Supportive Care in Cancer score [14], to indicate primary prophylaxis with growth colony stimulating factors or by patients regularly monitoring their temperature at home to seek emergency care when fever ensues [15]. Despite these existing methods, FN still has an important economic and clinical impact in cancer care. The early detection of neutropenia could be used to prevent FN by triggering an early administration of granulocyte colony-stimulating factor or antibiotics [16-19]. Unfortunately, current neutropenia-monitoring options rely on venipunctures in the clinical setting or finger-prick blood samples at the point of care [20]. These technologies either require laboratory infrastructure limited to the hospital setting or are impractical as they cannot be operated by minimally trained users to achieve accurate and reliable results [21-24]. To address this unmet need, this paper presents a usability evaluation of a novel, noninvasive technology that allows automated and frequent neutropenia monitoring by patients from the home setting with minimal training.

Assessing the usability for this kind of technology is crucial in ensuring the accuracy of the results, driving adoption, and improving patient compliance and adherence [25]. According to the International Organization for Standardization (IEC 62366-1:2015), usability is defined as a “characteristic of the user interface that facilitates use and thereby establishes effectiveness, efficiency and user satisfaction in the intended use environment” [26]. These metrics can be measured by gaining insight into patient perspectives regarding user performance, satisfaction, and acceptability while using an intervention [27]. For this study, the standardized System Usability Scale (SUS) survey was chosen as a quantitative method of assessing subjective usability due to evidence that it can be used to assess any technology [28] and has successfully been used in the medical domain to assess home medical devices [29-31].

The early detection of FN risk is essential as it can be associated with a higher chance of survival, more successful treatment, and improved quality of life. Therefore, the need for these technologies to be user friendly to the majority of the patient population subsequently increases, as this can impact the patients’ perception of the technology and their decision to take the test [32]. Additionally, technology-based solutions such as the one presented in this paper can help strengthen the relationship and communication between patients and their doctors, empower the patients’ well-being, and help doctors make better and more informed decisions [33].

### Study Objectives

We hypothesized that novice users will consider PointCheck (Leuko Labs), the first noninvasive optical technology for screening severe neutropenia, to be easy to use. The primary study objective was to evaluate the usability of PointCheck with the goal of identifying potential user interface (UI), functionality, and design issues from the perspective of untrained, first-time users in a simulated home environment. The primary end point for the study, defined a priori, was a group mean score of 80.8 on a standardized SUS, indicating a favorable perception of usability and a higher likelihood of adoption.

## Methods

### Device Description

**Figure 1:**
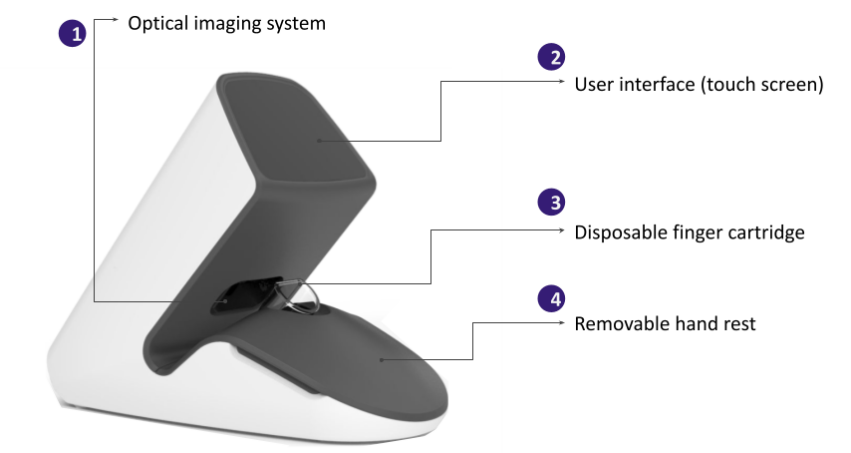
The PointCheck device and its main components.

PointCheck is the first noninvasive device (Figure 1) designed to screen for severe neutropenia in the home setting [34]. By imaging the blood flowing through the capillaries in the finger, PointCheck enables real-time remote monitoring of WBC levels based on optical imaging and without a blood draw [35,36].

The device consists of an optics and illumination system, on-board computing electronics, an 8.9-cm touch screen UI, a power cord, and disposable finger cartridges (Figure 1). It uses a camera microscopy system and LEDs to image capillaries in the nailfold region of the finger—typically the nondominant 4th (ring) finger, which has been shown by previous literature to contain the most intact and visible capillaries when compared to other fingers [37]. The finger cartridge is a disposable component that is prefilled with mineral oil and allows for effective optical refractive index coupling to ensure transdermal imaging quality [38,39]. The finger cartridge is designed for 1-time use. The hardware system design resembles the methods used in standard nailfold video capillaroscopy, which is an established technique used by rheumatologists to evaluate capillary morphology and microcirculation [40].

**Figure 2:**
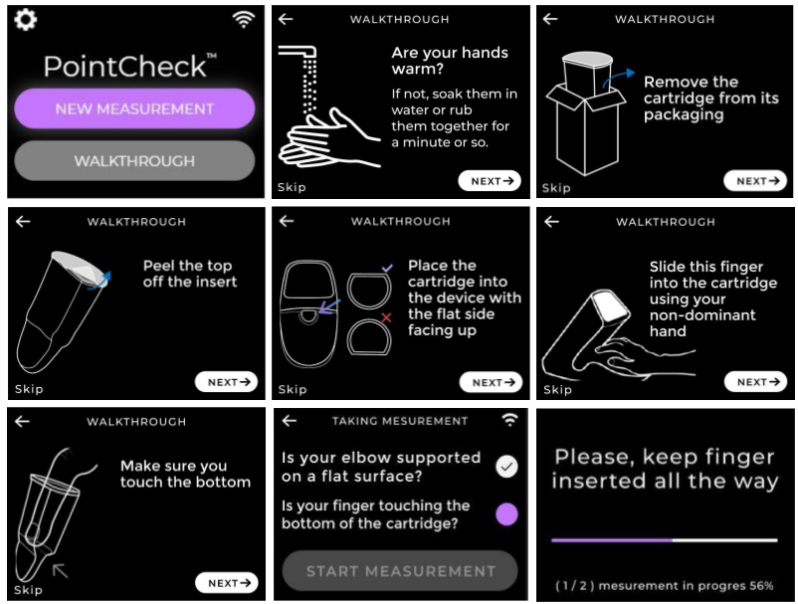
Screenshots of PointCheck’s user interface depicting the user walk-through tutorial in English for taking a measurement and device function via the touch screen interface. Language support for Spanish- and Haitian-speaking populations was implemented to translate the instructions.

The UI on the touch screen provides a guided walk-through to facilitate the correct use of the device. It prompts the user to warm their hands; open up a new, unused cartridge; properly place the cartridge into the device; and insert their nondominant 4th (ring) finger all the way into the cartridge while properly supporting their arm on a flat, stable surface (Figure 2). A final checklist ensures that the most critical steps have been completed and the user is able to start the 1-minute measurement. The version of the device used in this study was a beta prototype (version 4).

### Participants and Setting

Usability data was gathered from a cohort including both healthy volunteers and outpatients with cancer receiving chemotherapy. Patients were recruited at both the Boston Medical Center and Hospital Universitario 12 de Octubre before their routine chemotherapy administration. The healthy volunteers were recruited at the Massachusetts Institute of Technology’s Center for Clinical and Translational Research via advertisements displayed on Massachusetts Institute of Technology’s campus and social media and via email lists. The study visits took place in a simulated home environment, and testing was conducted without supervision from a medical professional. No participants had prior experience with the tested device.

A total of 154 participants (85 patients and 69 volunteers) participated in this study. According to standard usability sample size models, this sample size provides a 99% chance of detecting errors with the probability of occurrence of 3% at least once [41].

To ensure the generalizability of the results, we included younger (aged <65 years) and older (aged ≥65 years) adults, patients with diverse cancer types (lymphoma, leukemia, and myeloma, among other tumor types), men and women, and different education levels (≥8th grade or <8th grade). This allowed us to better understand the links between certain characteristics of the potential patients (ie, age, education, technophilia, and health literacy) and the usability [42,43].

### Ethics Approval

Institutional review board (IRB) approvals were obtained from the Boston Medical Center IRB (H-39964), Hospital Universitario 12 de Octubre IRB (20/049), and the New England IRB (1290027) to conduct the study prior to recruitment. Participants provided written consent before agreeing to participate in the study according to good clinical practice guidelines (ICH E6:R2) [44].

### Study Design

We used a mixed method approach to gather usability data through (1) user observation, (2) a short-answer qualitative e-questionnaire, and (3) a standardized quantitative SUS to assess perceived usability and satisfaction.

Regarding user observation, study coordinators observed participants while they used the device to document any errors that could potentially lead to imaging errors on the device. For example, an unsupported arm or incorrect hand placement could result in too much movement during a measurement and cause an error in the reading. Study coordinators also observed participants to identify and document any points of confusion during the walk-through steps that could be improved. All documented observations were collated into a list to be manually categorized by the type and frequency of occurrence (see Qualitative Results).

A subset (n=120) of the participants were given the opportunity to give feedback and document their thoughts, feelings, and experience using the device through an e-questionnaire containing 4 questions (Multimedia Appendix 1). We used this questionnaire to assess any potential confusion or difficulties participants may have had using the device or the UI, their attitude toward the product, and any potential features they would like to see added to improve user friendliness. The feedback from the questionnaires was collated into a spreadsheet to be manually categorized into themes (see Qualitative Results).

Finally, the SUS survey was used as a method of assessing subjective usability. The SUS is a Likert-type questionnaire comprising 10 questions with 5 response options ranging from “strongly disagree” to “strongly agree,” allowing for a subjective assessment of usability [45]. Scores range from 0 to 100 with higher scores indicating favorable user perceptions of the device and lower scores indicating low usability. The success criterion for a favorable evaluation in the SUS was defined by a mean group score of greater than 80.8 (see Quantitative Results). This threshold was selected based on previously published cutoffs to define the promoters of a technology [46].

### Usability Testing Procedure

**Figure 3:**
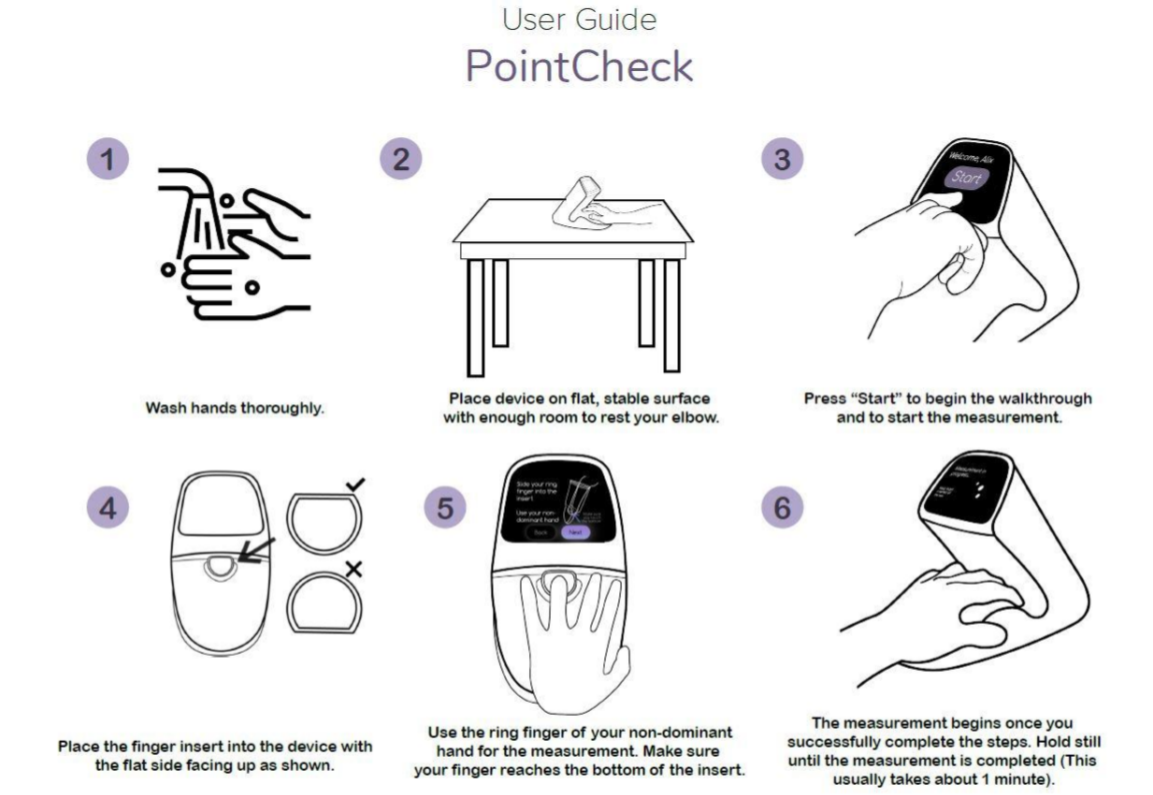
One-page quick start guide provided to participants before attempting to take a measurement on their own.

Baseline assessments conducted by research staff included a brief physical examination and collection of demographic information. Study coordinators read a short script that provided the participants with information about how to use the device and emphasized that the aim of the study was to test the user friendliness of the device and not the participants’ ability to use the device correctly (Multimedia Appendix 2). In addition, the participants were provided with a 1-page guide containing device instructions before attempting to take a measurement on their own (Figure 3).

Participants were then asked to follow the instructions presented to them on the device screen, guiding them through the critical steps required to obtain high-quality measurements. The study coordinators did not intervene or answer questions related to device use to reproduce the conditions of unsupervised home use. A second observer monitored and recorded a subset of visits either in person or through the Zoom teleconferencing platform (Zoom Video Communications). Observers documented participant errors, feedback, and tendencies. After completing the initial measurement, participants were immediately asked to complete the SUS and questionnaire to evaluate their first impressions about the user friendliness of the system to prevent any bias introduced from repeating measurements and becoming familiar with the measurement process. Participants performed additional trials, each lasting about 1.5 minutes (1-minute measurement plus 30-second setup and walk-through) for a total of 2 to 6 repeat measurements to evaluate the device precision. These subsequent trials were not used for the perceived usability evaluation and are not reported here.

### Data Analysis

Basic demographic characteristics were summarized using descriptive statistics. A final SUS score was computed in accordance with Brooke [47], and responses to the e-questionnaire were tokenized for their content and categorized into themes for qualitative analysis. Statistical comparisons were made between the different group categories stratified by age and literacy to evaluate usability differences using nonparametric techniques (Mann-Whitney *U* test). All quantitative data were processed using RStudio (version 1.3.1093; RStudio Team) [48].

## Results

### User Statistics

Table 1 represents the breakdown of study participant characteristics by age, education level, gender, and race. Of the 154 participants, 118 (76.6%) were aged <65 years, with an average age of 44.8 (range 18-88) years. A majority (n=102, 66.2%) of the participants had an education level exceeding 8th grade, whereas 43 (27.9%) participants had an education level below 8th grade, and 9 (5.8%) did not provide educational level information.

**Table 1:** Basic demographics of participants. Educational, race, and ethnicity level data were missing for 9 (5.8%), 11 (7.1%) and 10 (6.5%) out of 154 participants, respectively.

Demographic		Participants (N=154)
**Age (years)**		
	Mean (SD)	44.8 (20.5)
	Median (range)	38.3 (18.0-88.5)
**Gender, n (%)**		
	Male	67 (43.5)
	Female	87 (56.5)
**Educational level, n (%)**		
	<8th grade	43 (27.9)
	≥8th grade	102 (66.2)
	Missing	9 (5.8)
**Race, n (%)**		
	American Indian or Alaska Native	1 (0.6)
	Asian	28 (18.2)
	Black or African American	28 (18.2)
	More than 1 race	5 (3.2)
	Unknown	11 (7.1)
	White	81 (52.6)
**Ethnicity, n (%)**		
	Hispanic or Latino	49 (31.8)
	Not Hispanic nor Latino	95 (61.7)
	Unknown	10 (6.5)

### Quantitative Results

**Figure 4:**
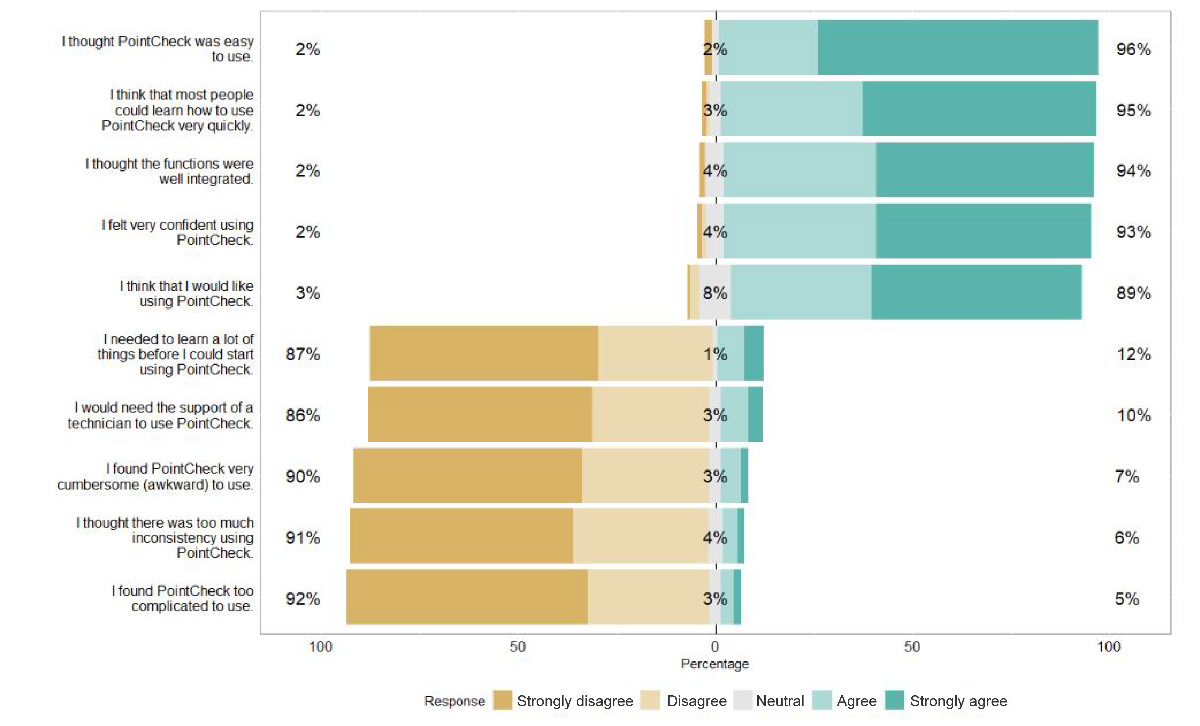
SUS survey responses assessed individually. Percentage values are calculated using available data from a total of 151 participants. SUS surveys were incomplete for 3 participants. SUS: System Usability Scale.

The average SUS score across all participants was 86.1. In total, 70.1% (108/154) of the participants scored above the goal of 80.8 (Tables 2 and 3), which indicated that they would be early promoters and more likely to recommend the device to a friend [49]. When stratifying the SUS results by education level, we found that participants exceeding the 8th grade level scored slightly higher than those with an 8th grade level education and below—but only by a margin of 2.5 points, which was not found to be statistically significant (*P*=.27; Table 4). When stratifying SUS results by age categories, we found that participants aged <65 years also scored higher than participants aged ≥65 years by a margin of 3.4 points, showing a nonstatistically significant trend (*P*=.06). Both groups had a mean score above the predefined threshold of 80.8 (Table 5). When evaluating the SUS results by the individual survey questions, we found that 96% (145/151) of the participants that completed the survey found PointCheck easy to use and felt that they could learn to use it very quickly (Figure 4).

**Table 2:** Quantitative System Usability Scale (SUS) results across all participants. SUS surveys were incomplete for 3 participants and could not be computed.

SUS score (range 0-100)	Overall (N=154)
Mean (SD)	86.1 (12.2)
Median (range)	87.5 (20.0-100)
Missing, n (%)	3 (1.9)

**Table 3:** Total percent of promoters (defined as participants scoring >80.8 points on the System Usability Scale). System Usability Scale surveys were incomplete for 3 participants and could not be computed.

Cutoff used (points)	Overall (N=154), n (%)
≤80.8	43 (27.9)
>80.8	108 (70.1)
Missing	3 (1.9)

**Table 4:** System Usability Scale (SUS) results stratified by educational level (<8th grade and ≥8th grade). The results were generated using the data available from a total of 145 participants. Educational level data was missing for 9 participants (N=154, 5.8%).

SUS score (range 0-100)	<8th Grade (N=43)	≥8th Grade (N=102)	Overall (N=154)
Mean (SD)	84.0 (12.7)	86.5 (12.2)	86.1 (12.2)
Median (range)	85.0 (57.5-100)	87.5 (20.0-100)	87.5 (20.0-100)

**Table 5:** System Usability Scale (SUS) results stratified by age category (<65 years and ≥65 years).

SUS score (range 0-100)	<65 years (N=118)	≥65 years (N=36)	Overall (N=154)
Mean (SD)	86.9 (12.5)	83.5 (11.0)	86.1 (12.2)
Median (range)	90.0 (20.0-100])	82.5 (57.5-100)	87.5 (20.0-100)

### Qualitative Results

#### User Observation

Error observation notes were collated into a list and then manually categorized by the type and frequency of occurrence. The primary error sources included skipping or misreading instructions and on-screen instruction accessibility. The majority (70/86, 81%) of these errors occurred only on the first use and were shown to be correctable by interventional guidance. Such guidance was given after the SUS survey had been completed, and improvement in most cases was demonstrated in subsequent trials. This shows that although the device performs well in independent use, the monitoring of first use by an experienced operator may have further benefit for catching usability errors.

#### e-Questionnaire Feedback

The feedback from the questionnaires was collated into a spreadsheet and then manually categorized into the following themes: UI/user experience, aesthetic design/logical design, hand rest, cartridge, cleaning/sanitation, and software/bugs. The themes were then broken down into the following subthemes: confidence in use, training effectiveness, UI design/clarity of UI instructions, ergonomic design, foreseeable home use issue, and accessibility. The instances of feedback falling within these subthemes were counted and generated the 3 overarching themes: pretraining effectiveness, user friendliness of PointCheck (related to ease of use, accessibility, and clarity of UI elements), and ergonomic design.

#### Pretraining Effectiveness

A portion of participants initially expressed some uncertainty when using the device for the first time (Participants #38 and #18; Table 6). With repeated use, however, most participants felt that they could catch on quickly (Participant #40; Table 6).

**Table 6:** Illustrative quotes for the 3 overarching themes.

Theme/category	Illustrative quote
Pretraining effectiveness	“Would like a YouTube channel/clip to watch in advance that will explain the device.” (Participant #38) “The most difficult step was probably removing the cartridge from the box. I was not sure if I had to keep the cartridge clean for measurements and the instructions did not tell how I should be holding the cartridge or if I should even be careful or not about touching it too much and getting it dirty” (Participant #18) “The device was cumbersome (awkward) to use because it was the first time. After the first time, it would be easier to use.” (Participant #40)
User friendliness of PointCheck	“Others can’t see as well, may need others to help them if they have dementia or are forgetful.” (Participant #53) “Could not read font of the three step instructions.” (Participant #65) “If I were to use the device on a daily basis, I would be relatively annoyed by the fact the three repeat questions are timed lag to press yes.” (Participant #15) “One thing I was confused about was checking off ‘was my finger in all the way.’ It was just a circle and I was confused what I was supposed to do on this step.” (Participant #21) “The cartridge lid is shown to be peeled from the flat side (facing down) but then needs to be rotated to be put in with the flat side up. If the peel could be opened in the right orientation, that would have helped.” (Participant #24) “Very crisp, and clear how to start using it...the screen has good contrast, good font choice given resolution and size, and the purple/grey color scheme is also calming.” (Participant #30) “Even though I knew beforehand that I should rest my elbow, I forgot to do so after inserting my finger because that was my main focus, so I really appreciated the reminder to have my elbow rested right after the step where I inserted my ring finger.” (Participant #34)
Ergonomic Design	“The display seems angled a little high, considering that the machine needs to be placed a considerable distance to rest my arm. The steps were fairly intuitive.” (Participant #3) “I was expecting the device to be a bit smaller. I think the device is designed for a very big hand, I think it would be probably better to try to make it the size closer to a computer mouse.” (Participant #16) “My fingers are pretty small and narrow but the soft spiked insides of the capture cylinder still left marks on my finger afterwards. This was completely non-painful but just noting this here for other users who might have thicker fingers! It was a bit bigger than I expected (the size of the device) but it doesn’t impact usability. It also produced quite a loud hum but again, doesn’t impact usability.” (Participant #11) “I liked the brush-like texture inside the tube. It helped me feel that I had my finger in the right position.” (Participant #33) “My first impression was that it looked pretty compact and that it’s very straight to the point in its features--nothing fancy, just functional.” (Participant #34)

#### User Friendliness of UI Design

Older participants (aged ≥65 years) discussed the need for improved screen readability, mentioning increasing the font size or needing additional assistance (Participants #53 and #65; Table 6).

There were mixed opinions on the overall design of the UI, but the majority (145/151, 96%) of the participants found the overall system to be easy to use and that they could learn quickly. Some participants did comment on the elements of UI design, such as buttons, on-screen instructions, or color choices, that made them feel frustrated, confused, or uncertain about whether they were performing the measurement correctly (Participants #15, #21, and #24; Table 6). Other participants expressed satisfaction with the UI design (Participants #30 and #34; Table 6).

#### Ergonomic Design

Finally, participants also addressed the changes they wished to see in the ergonomic design to better meet the needs of end users (Participants #3, #16, and #11; Table 6). Other participants expressed satisfaction with the ergonomic design (Participants #33 and #34; Table 6).

## Discussion

In this study, we aimed to evaluate the usability and design of PointCheck, a novel technology for noninvasive, home-based neutropenia detection. Through a mixed method approach of user observation, questionnaires, and a SUS survey, we have validated the hypothesis that PointCheck is easy to use by first-time users in a simulated home environment with a mean SUS score of 86.1 (Table 2), classified as a score of A (ie, excellent; net promoter score: promoter level) [50,51] and falling within the top tenth percentile of systems as evaluated by the SUS [49].

Although the majority of first-time users expressed high satisfaction with the overall design and user friendliness of PointCheck (Table 3), a number of areas for improvement were identified through the feedback and observation of users and will be implemented into future designs. The main changes to be implemented to enhance the usability of the device and reduce instances of errors include the addition of a tutorial video and walk-through image animations as additional training methods, improved screen readability, improved button design to make them easily identifiable to users, and a modification of the cartridge to be more size inclusive.

In observing the use of the device in context, correct positioning during the use of the device may be more difficult for nonambulatory patients. Ideally, patients will have a training session with their health care professional prior to bringing this device home for normal use. This training would allow patients to familiarize themselves with the device beforehand, ask any questions related to use, and receive the support needed to ensure confidence in using the device alone for weeks at a time.

It is to be noted that a majority of study participants were aged <65 years and have an educational level of ≥8th grade level, both of which are factors that increase the likelihood of technological proficiency and willingness to adopt new technology [52]. Emerging technologies such as smartphones and tablets have raised concerns about their ease of use in older and untrained populations [53]; however, we found that it did not affect the perceived usability of PointCheck, considering that there was no significant differences in the SUS scores among users across educational levels and age categories (Tables 4 and 5). This is consistent with prior literature which has demonstrated that older populations are interested and capable of using modern technologies for managing health and can learn how to use a touch screen after a few tries [53,54]. Although all participants in the study had no previous training or experience using PointCheck, all of them were able to become proficient after 1 or 2 measurements guided by experienced clinicians through repetition by the end of the study visit. This is an indication that training, while necessary for building intuition and confidence when using the device [55], does not need to be extensive and the on-screen walk-through is effective in guiding the user through a measurement alone.

Although this study aimed to evaluate a variety of usability factors in a simulated home environment, a single study cannot claim to assess these factors in all use cases and situations. The perceived usability of PointCheck should be tested further in real-world home environments with users who receive prior training to identify context-related issues in the future.

Overall, this study demonstrated that PointCheck, a novel digital device for noninvasive WBC monitoring, can be easy to use for unsupervised patients in the home setting. By enabling continuous home-monitoring for severe neutropenia, PointCheck has the potential to change the standard of care for patients with cancer and substantially improve their clinical outcomes.

## Appendix

Multimedia Appendix 1 Short-answer qualitative e-questionnaire.

Multimedia Appendix 2 Study coordinator training script.

## Abbreviations

FN: febrile neutropenia
IRB: institutional review board
SUS: System Usability Scale
UI: User Interface
WBC: white blood cells
