# Supplementary material for: Usability Evaluation of a Noninvasive Neutropenia Screening Device (PointCheck) for Patients Undergoing Cancer Chemotherapy: Mixed Methods Observational Study
Source: J Med Internet Res. 2022 Aug 9;24(8):e37368. doi: 10.2196/37368 (PMC9621111; doi:10.2196/37368)
Supplement: Multimedia Appendix 1 [file jmir_v24i8e37368_app1.pdf]

**Appendix 1: Short Answer Qualitative E-Questionnaire**

1. What was your first impression of this device?
2. What was challenging about this device?
3. What would you say was the most difficult step in using this device?
4. If I sent this device home with you and asked you to use it by yourself without any help or supervision, would you see any difficulties in using it? Like what?
