# Supplementary material for: Usability Evaluation of a Noninvasive Neutropenia Screening Device (PointCheck) for Patients Undergoing Cancer Chemotherapy: Mixed Methods Observational Study
Source: J Med Internet Res. 2022 Aug 9;24(8):e37368. doi: 10.2196/37368 (PMC9621111; doi:10.2196/37368)
Supplement: Multimedia Appendix 2 [file jmir_v24i8e37368_app2.pdf]

Appendix 2: Study coordinator training script

Hi,________. My name is ________, and I'm going to be walking you through this session.

You probably already know, but let me explain why we've asked you to come here today: We're testing a new non-invasive diagnostic device that we're working on to see what it's like for actual people to use it. It shines a light on your skin to view your nailfold capillaries and white blood cells using a microscopic camera.

I want to make it clear right away that we're testing the user friendliness and function of the device, not you. You can't do anything wrong here. In fact, this is probably the one place today where you don't have to worry about making mistakes.

We want to hear exactly what you think, so please don't worry that you're going to hurt our feelings. We want to improve it, so we need to know honestly what you think.

As we go along, I'm going to ask you to think out loud, to tell me what's going through your mind as you use the device and follow the on-screen walkthrough. This will help us.

If you have questions, just ask. I may not be able to answer them right away, since we're interested in how people do when they don't have someone sitting next to them, but I will try to answer any questions you still have when we're done.

This is the PointCheck device and this is the “cartridge” box. For this study, we will have you operate the device yourself and collect a total of two measurements. First, I will have you watch a demonstration video [click on video on home page]. I’ll need you to sit comfortably in this chair in a way that will allow you to rest your elbow on a flat, stable surface. Then you can start the measurement process by using the touch screen and following the instructions.

Do you have any general questions before we begin?
